# Protein Predictive Modeling and Simulation of Mutations of Presenilin-1 Familial Alzheimer’s Disease on the Orthosteric Site

**DOI:** 10.3389/fmolb.2021.649990

**Published:** 2021-06-02

**Authors:** Alejandro Soto-Ospina, Pedronel Araque Marín, Gabriel Bedoya, Diego Sepulveda-Falla, Andrés Villegas Lanau

**Affiliations:** ^1^ Faculty of Medicine, Group Molecular Genetics, University of Antioquia, Medellín, Colombia; ^2^ Faculty of Medicine, Group Neuroscience of Antioquia, University of Antioquia, Medellín, Colombia; ^3^ School of Life Sciences, Research and Innovation in Chemistry Formulations Group, EIA University, Envigado, Colombia; ^4^ Molecular Neuropathology of Alzheimer’s Disease, Institute of Neuropathology, University Medical Center Hamburg-Eppendorf, Hamburg, Germany

**Keywords:** presenilin-1, modeling, simulation, quantum mechanics/molecular mechanics, familiar Alzheimer’s disease mutations

## Abstract

Alzheimer’s disease pathology is characterized by β-amyloid plaques and neurofibrillary tangles. Amyloid precursor protein is processed by β and γ secretase, resulting in the production of β-amyloid peptides with a length ranging from 38 to 43 amino acids. Presenilin 1 (PS1) is the catalytic unit of γ-secretase, and more than 200 PS1 pathogenic mutations have been identified as causative for Alzheimer’s disease. A complete monocrystal structure of PS1 has not been determined so far due to the presence of two flexible domains. We have developed a complete structural model of PS1 using a computational approach with structure prediction software. Missing fragments Met1-Glut72 and Ser290-Glu375 were modeled and validated by their energetic and stereochemical characteristics. Then, with the complete structure of PS1, we defined that these fragments do not have a direct effect in the structure of the pore. Next, we used our hypothetical model for the analysis of the functional effects of PS1 mutations Ala246GLu, Leu248Pro, Leu248Arg, Leu250Val, Tyr256Ser, Ala260Val, and Val261Phe, localized in the catalytic pore. For this, we used a quantum mechanics/molecular mechanics (*QM/MM*) hybrid method, evaluating modifications in the topology, potential surface density, and electrostatic potential map of mutated PS1 proteins. We found that each mutation exerts changes resulting in structural modifications of the active site and in the shape of the pore. We suggest this as a valid approach for functional studies of PS1 in view of the possible impact in substrate processing and for the design of targeted therapeutic strategies.

## Introduction

Neurodegenerative diseases are characterized by impairment of the central nervous system (Bereczki et al., 2018). Many of these pathologies are produced by deposits of proteins as Huntingtin in the case of Huntington disease (HD), α-synuclein for Lewy body in Parkinson’s disease (PD), neurofibrillary tangles by hyperphosphorylation of tau (τ) protein, and senile plaques by accumulation of β-amyloid (Aβ) peptide in Alzheimer’s disease (AD) (Myers, 2004; Paulsen, 2011; Jucker and Walker, 2012; Jucker and Walker, 2013). AD is the most common of neurodegenerative diseases, representing the largest number of reported cases worldwide (Sheikh et al., 2013; Prince et al., 2015).

AD must be divided into familiar AD (FAD) and sporadic AD (SAD). FAD is caused by the inheritance of mutations in the genes for amyloid precursor protein (APP), presenilin-1 (PSEN1), and presenilin-2 (PSEN2), and it can manifest in different ages (Rovelet-Lecrux et al., 2006; Guerreiro and Hardy, 2014; Shao et al., 2017). SAD is thought to be associated with known risk factors for other diseases, for example, high-cholesterol blood levels (dyslipidemia), oxidative stress, inflammation, low cognitive activity, and absence of physical activity (Ballard et al., 2011; Arbor et al., 2016; Yu and Zheng, 2012). The amyloidogenic theory states that AD is the result of the accumulation of Aβ, a fragment of the APP protein. APP metabolism follows two pathways, a non-amyloidogenic and an amyloidogenic pathway. In the non-amyloidogenic pathway, the enzyme α-secretase cleaves APP followed by cleavage by γ-secretase, a transmembrane protein complex, producing a small peptide of 23 amino acids (peptide p3) and an intracellular fragment known as the APP intracellular domain (AICD) (Lichtenthaler, 2012; Eggert et al., 2004). On the other hand, in the amyloidogenic pathway, APP is cleaved by β-secretase first and then by γ-secretase, producing Aβ peptides of diverse length (1–38 to 1–43 amino acids) (Vassar, 2004; Venugopal et al., 2008; Chávez-Gutiérrez et al., 2012). Aβ peptide structure facilitates its oligomerization, resulting in the accumulation of senile plaques in brain parenchyma (Wolfe et al., 1907), (Thal et al., 2008). The γ-secretase enzymatic complex includes four subunits: presenilin-1 (PS1), pharynx-defective 1 (Aph1), nicastrin (NCT), and presenilin enhancer-2 (PEN-2). PS1 is the catalytic unit of the γ-secretase complex, and its orthosteric site is located in aspartic acids 257 and 385, in transmembrane helix 6 and 7, respectively (Chávez-García et al., 2019). PS1 contains low mobility regions including nine α-helixes, and two high mobility regions, so far without a defined structure (Wolfe et al., 1907; Cacquevel et al., 2012; Bai et al., 2015). Only recently, a comprehensive structural analysis of PS1 was possible thanks to protein crystallization and cryogenic electron microscopy (cryo-EM). However, in order to obtain a crystal structure, flexible domains, such as amino acids Met1 to Glu 72 and Ser 290 to Glu 375, were not included in the sequence (Zhou et al., 2019).

The lack of a full crystal structure for PS1, including high-mobility regions, makes difficult to explain the possible role of some mutations, their impact on neuronal pathology, and it hinders the development of effective medical treatments. Moreover, protein loops can have special functions, including domain recognition and regulatory activities. For instance, PS1 loops seem to be responsible of the activation of the catalytic function (Wolfe et al., 1907; Knappenberger et al., 2004; Fukumori et al., 2010).

In order to provide a more precise correlation between PS1 structure and gamma secretase function, it is important to determine the localization and the 3D structure of PS1 missing fragments, using other tools such as bioinformatics and structural modeling. In this work, we have used three different predictive algorithms in order to complete the structural 3D model for PS1.

The dynamic methods that are part of macromolecular systems consist of computational simulations of particles in movement. Molecular dynamics utilize special algorithms to explain motion states and geometrical conformations for systems where several forces are acting simultaneously at various magnitudes of interactions and angles. These are always based on the classic Newtonian physic principles, but under rigid charge distribution (Nosé, 1984; Hospital et al., 2015). There are several structural models proposed in literature for the γ-secretase enzyme (Bai et al., 2015; Zhou et al., 2019; Bai et al., 2015). These are based on the role of protonation and deprotonation of the aspartic acids Asp257 and Asp385, in the substrate immobilization around the pore (Aguayo-Ortiz and Dominguez, 2018; Hitzenberger and Zacharias, 2019; Bhattarai et al., 2020). Additionally, it considers the ability of the enzyme to recognize the extracellular APP which contains the subunit NCT (Bolduc et al., 2015), which is in charge of constraining the substrate by means of hydrogen bonding. All these molecular effects considered in the whole molecular dynamic, along with the configurational arrangements, result in a rigid secondary structure of the enzyme (Hitzenberger and Zacharias, 2019; Aguayo-Ortiz et al., 2017). It is important to consider as well, the effect of all possible mutations of the PS1 that occur far from the active site, with the supporting proteins PEN2 and APH1 which can be modulated according to the protonation degree of the orthosteric site and evidencing that these simulations do not take into account a complete model of the catalytic subunit PS1, to represent the missing fragments of the protein and the correlation of various electronic effects (Chávez-García et al., 2019).

Several hybrid methods in quantum mechanical molecular field have been widely utilized for the study of the macro biological systems. These allow to register and quantify small changes that the enzyme undergoes, considering polarizable electrons as the most susceptible to the measurements of the missense-nonsynonymous variants with quantum methods such as functional density implementing the B3LYP or even the Hartree–Föck method (Murphy et al., 2000; Orlando and Jorgensen, 2010; Náray-Szabó et al., 2013; Roston et al., 2018; Siegbahn and Blomberg, 2018) to calculate the reaction coordinate and the formation or breaking of chemical bonds. These present some disadvantages, principally due to the limited amount of nucleus and electrons considered for a given biological system. Besides, these compute complex matrices that require high computational resources. On the other hand, semi-empirical methods seem to be a promising alternative in the study of biological and polyatomic systems as these intend to solve the Schrödinger equation from an approximated perspective, that is, considering an average between the electron interactions and appropriated theory levels, reducing the computational time. Of particular interest, the semi-empirical Austin model 1 is characterized because its parameters are derived from experimental data in order to solve the Schrödinger equation (Carvalho et al., 2014; Christensen et al., 2016; Rafique et al., April 2019; Garcia et al., 2020; Grillo et al., 2020). These can be applied efficiently to large molecules to calculate their respective surface potentials (Foresman and Frisch, 1996; Levitt, 2014; Silva et al., 2015; Marín and Soto-ospina, 2020; Cano et al., 2021).

In this work, we have analyzed the structural changes in the active site resulting from seven selected mutations in TM6 and TM7 of PS1, utilizing a hybrid method of quantum mechanics/molecular mechanics simulation. We have encountered that once a full 3D model for PS1 is achieved. Furthermore, the conformational effects of mutations Ala246GLu, Leu248Pro, Leu248Arg, Leu250Val, Tyr256Ser, Ala260Val, and Val261Phe, localized in the pore, can be explained as polarity changes, torsion angles, distance between helixes, and electronic structures. We proposed this analytical approach as the tool of choice for assessing mutational effects in structurally defined regions within proteins with multiple possible mutations in the nonflexible zones of PS1.

## Methodology

### Characterization of Protein PS1 Transmembrane Domains

A preliminary study of the structure of protein PS1 was performed by constructing a plot with the tool TMHMM available in the suite ExPASy (Sonnhammer and Krogh, 1998; Krogh et al., 2001; Möller et al., 2001), based on the primary sequence of the protein. We obtained a plot for the probability distribution through Hidden Markov Models. The position and score of the transmembrane fragments containing the structure inside–outside of cellular membrane were also determined (Artimo et al., 2012; Guex and Peitsch, 1997).

### Missing Fragments Structural Prediction and Characterization of Obtained Models

Three different software tools for structure prediction were used to build a hypothetical model of the fragments representing the missing loops of PS1. The crystallized structures for PS1 reported in the protein data bank (PDB) were chose with IDs: 6IYC, 5A63, and 5FN2 subunit B (Presenilin-1) (Bai et al., 2015; Zhou et al., 2019; Bai et al., 2015). The two missing fragments were identified, and the primary sequence was built with a hypothetical model, using an algorithm that was defined to create the models based on homology constitution. The protein’s active site was modeled based on the primary sequence of PS1 in FASTA format, and with the templates identified as 5A63 and 5FN2, which have aspartic acid in position 385 (D385) (Bai et al., 2015; Bai et al., 2015). The software tools used for the modeling were I-TASSER from Zhang Lab from the University of Michigan (Yang et al., 2015; Roy et al., 2010; Zhang, 2008) and Phyre2 (Protein Homology/analogY Recognition Engine V 2.0) from the structural bioinformatics group at Imperial College London (Kelly et al., 2015; Kelley and Sternberg, 2009). The models were refined with tools of the suite I-TASSER (Iterative Threading ASSEmbly Refinement), mainly using ModRefiner to optimize the energy from a native structure state and to improve the model for the interaction of backbone with hydrogen bond considering stereochemical optimization of the flexible behavior of the system (Xu and Zhang, 2011). Another refining tool used was the Fragment Guided of Molecular Dynamics (FG-MD). This software begins with classical modeling taking into account the geometrical optimization of angles and removing features that generated an unstable model. These features can improve steric clashes, geometry, and interactions by hydrogen bonding (Zhang et al., 2011). To follow other methodologies of elucidation with *de novo*, the software QUARK was utilized (or ran). This software is a tool for predicting a 3D model from each amino acid. This takes into account folded fragments of peptides and connects them via Monte Carlo simulation considering force fields and without utilizing templates (Xu and Zhang, 2012).

### Validation and Minimization of Predicted Models

Each model was validated with an energetic tool from the suite SIB EXpASY, using Z-score values and QMEAN6 for the assessment (Petrey et al., 2003; Soni and Madhusudhan, 2017). Then, the stereochemical distribution was characterized using the EMBL-EBI Procheck software (Laskowski et al., 1993). Ramachandran plots for measuring the dihedral planes between the residues of the peptide bonds in the protein constitution were obtained and a calculation for the angles Phi and Psi in the model was performed. Rampage software from Cambridge University for Ramachandran plot analysis was used for measuring the same angles using another algorithm (Artimo et al., 2012; Petrey et al., 2003; Ramachandran et al., 1963; Crystallography and Bioinformatics Group, 2017). All models were visualized with the software Chimera UCSF version 1.1.1 and aligned through the algorithm 3D by match-maker under the Needleman–Wunsch algorithm. A BLOSUM62 matrix was used for the global alignment of the PS1 protein (Pettersen et al., 2004). Structural minimization was simulated using packages NAMD—Scalable Molecular Dynamics and VMD—Visual Molecular Dynamics, for the elimination of bad initial contacts, to avoid overlapping, and to generate fluidity in the models generated (Phillips et al., 2005; Humphrey et al., 1996). The loops for the best final model for the complete PS1 protein were finally refined with the Modeller software tool. This tool generates a normalized value of discrete and optimized protein statistical potential for the best rotamers in the lateral chain. This is implemented under iterative cycles that consider possible spatial restrictions (Webb and Sali, 2016). Eventually, transversal views of modeled PS1 proteins were obtained using Chimera UCSF v.1.11.

### Hydropathicity Index and Phosphorylation Sites Prediction

The analysis of the polarity in the systems was performed with the software ProtScale available in the suite ExPASy, using as measurements the Kyte and Doolittle coefficients. Also, the primary sequences of the wild-type PS1 protein and studied mutants were assessed, and the hydropathy index was calculated for each amino acid, labeling it as hydrophobic or hydrophilic (Gasteiger et al., 2005). The NetPhos 3.1 Server was used to predict serine, threonine, or tyrosine phosphorylation sites susceptible to phosphorylation by diverse kinases, for instance, PKA, PKC, PKE, RSK, EGFR, or MAPK38 kinases (Artimo et al., 2012; Blom et al., 2004; Blom et al., 1999).

### Hybrid Method for Quantum Mechanics and Molecular Mechanics Modeling

**FIGURE 1:**
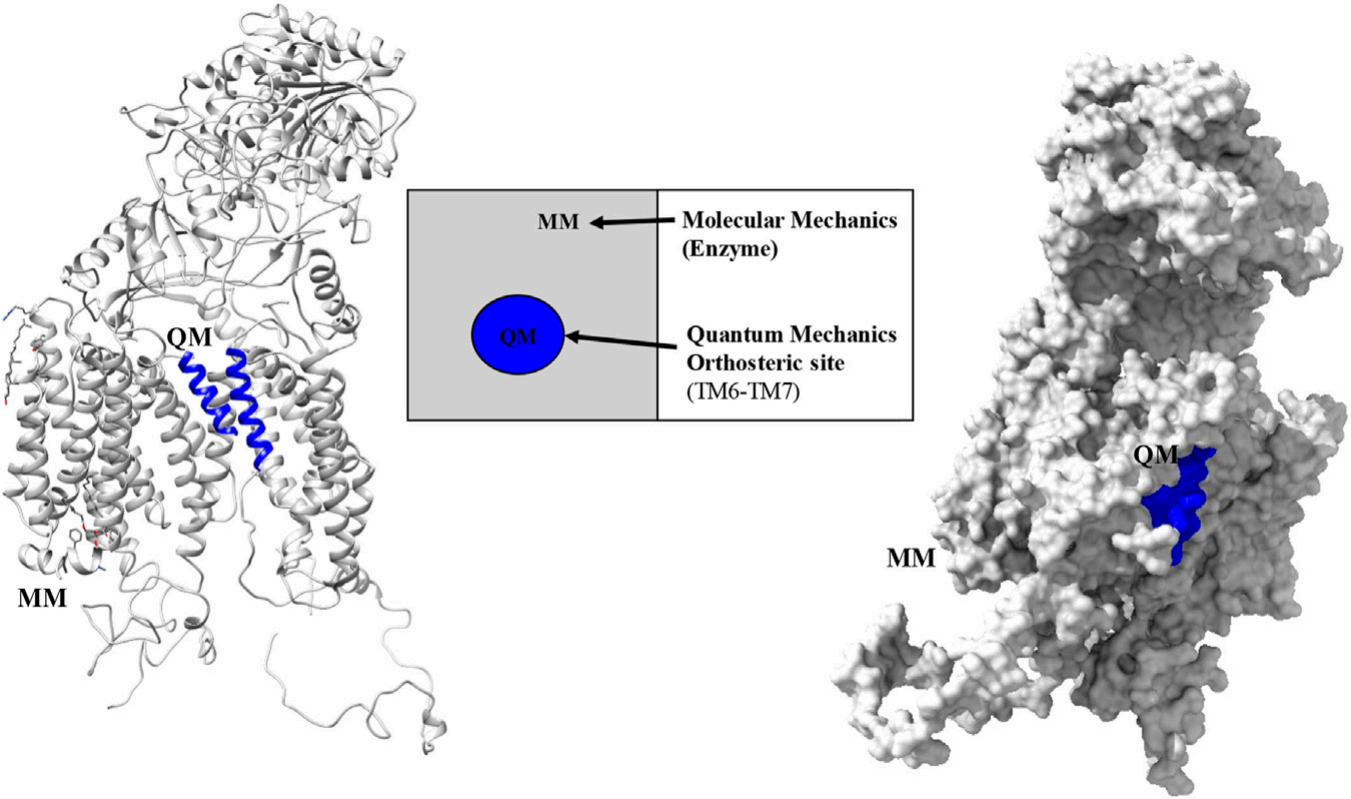
Partition regions observed with the QM-MM hybrid method for PS1 protein in the γ-secretase enzyme.

The final modeled structure of PS1 protein was used to study the functional behavior of mutations located in TM6: Ala246GLu, Leu248Pro, Leu248Arg, Leu250Val, Tyr256Ser, Ala260Val, and Val261Phe. The hybrid method examines the system based on the z-matrix obtained from the topological consideration for each nucleus, frozen for molecular mechanics purposes. The modeled system used the localization of α-helix TM6 amino acids 243–263 and TM7 383–398 as a representation of the active site. Then, a quantum mechanics calculation was applied to it, with a level of theory that consider the number of atoms and parametrization of the system, using data derived from experimental analysis published in databases for protein structure. However, the selected subsystem for implementing molecular mechanics (MM) observes the whole structure within a classical physics-based description of the remaining PS1 protein. The QM/MM fragment is considered with QM polarization due to the classical MM region for the TM6 and TM7 α-helixes, as shown in (Figure 1). The specific method used for this analysis was semi empirical applying the force field Austin model 1 (AM1) to the QM region (Dewar et al., 1993). This method considers the average interaction among electrons to solve the Schrödinger equation in protein macro systems. This method for geometric optimization is very useful given that the study of the canonical function considers 243–261 amino acids for region TM6 and 383–298 amino acids for region TM7. These have, in total, 280 atomic nuclei and 1,560 electrons for electronic description. Considering this, high-level quantum theory methods cannot be easily applied given the required computational resources and the costs of the calculation. The system’s total MM region and the classical description of the QM region is carried out with the MMFF(aq) by estimating the system’s second solvation sphere. The interface was also saturated with hydrogen atoms (Marques et al., 1995; Halgren, 1996; Halgren, 2000; Mackerell, 2004; Alexeev et al., 2013; Zhou et al., 2019; Soni et al., 2020). The molecules were optimized based on the data of global minimum geometry and energy using the Spartan 18′ software for wave function. This software has a tool for the determination of Spartan surfaces that simulate the optimized structure for each surface, such as density, potential–potential, ionization, orbitals Homo, orbitals Lumo, and electrostatic potential map. All of them resulted from the structural changes in the active site and applied to the electronic structure and their possible interactions.

The total energy in the system is calculated with the equation for mechanical integration:

$$E_{\text{total}}=EQM+EMM_{\left(\text{total}\right)}-EMM_{\left(QM\right)},$$ (1)

with this equation and under a multiscale analysis, the total energy is obtained for wild type and mutations models, tripled for each one, considering the average energy to be included in Eq. 1, and with a low standard deviation for each one of the determined systems (Maseras, 1999; Cao and Ryde, 2018).

## Results and Discussion

### Modeling of Loop Fragments of Protein PS1

**FIGURE 2:**
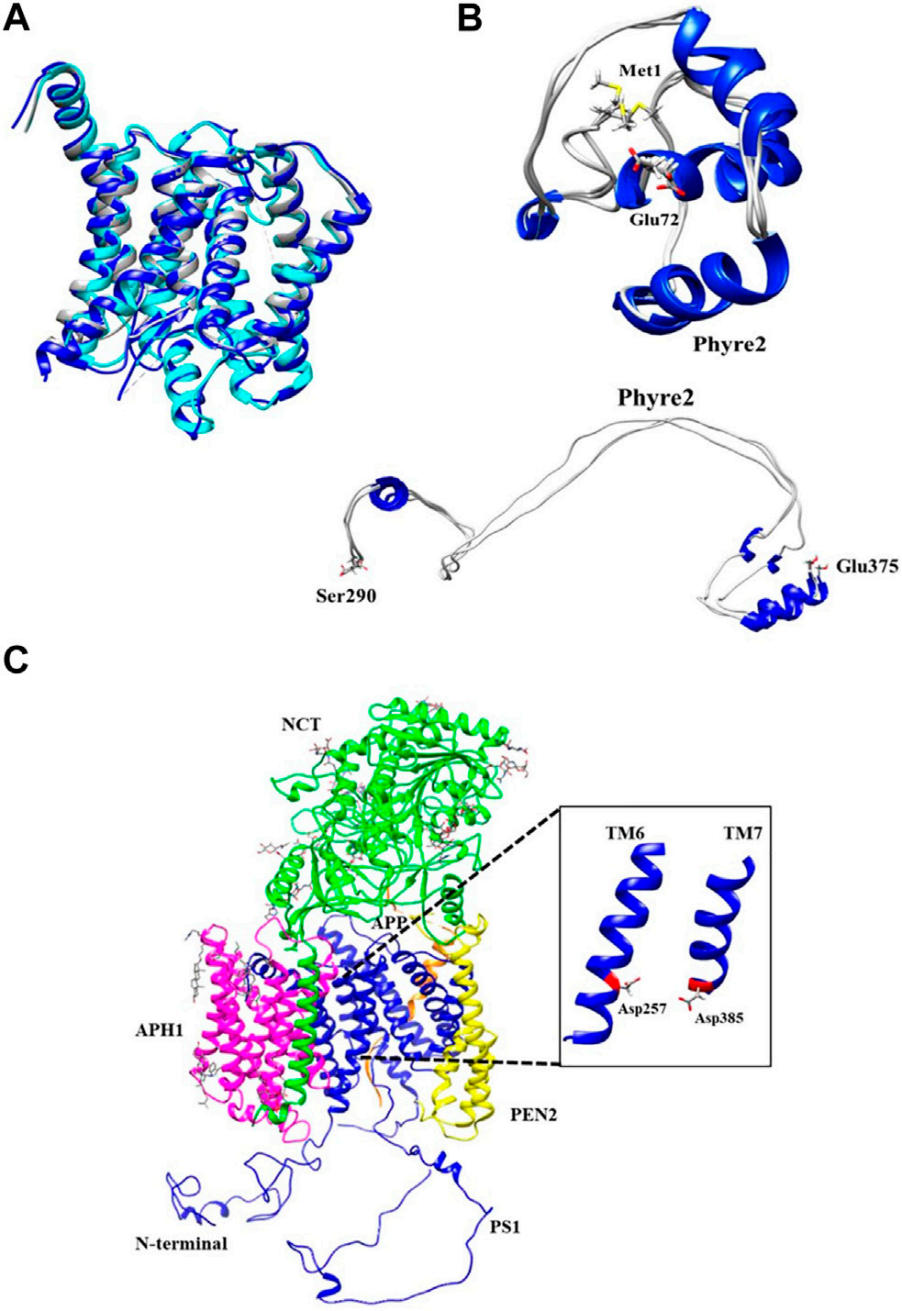
Phyre2 modeling for PS1 structure in the γ-secretase complex. **(A)** Structural alignment of cryo-electron microscopic structure PS1 protein domains as found in PDB ID: 6IYC (Blue), 5FN2 (Cyan), and 5A63 (Gray). **(B)** Refined and unrefined modeling of PS1 N-terminal fragment Met1-Glu72 and Ser290-Glu375, using Phyre2. **(C)** Modeling of the active γ-secretase heteromer. Inset shows PS1 transmembrane domains forming the pocket including TM6 and TM7 together with the active sites Asp257 and Asp385.

The PS1 structural template used as a baseline for the full structure prediction, was the one published by Zhou et al., Protein Data Bank (PDB) ID: 6IYC, given that it is the most complete structure up to date. Moreover, this structure was aligned with two other structures reported previously PDB ID: 5FN2 and PDB ID: 5A63 (Lu et al., 2014; Bai et al., 2015). Some minimum structural differences were found between the three models (Figure 2A). Missing fragments were incorporated as dotted lines. The specific amino acid position of the missing fragments was determined using the tool “sequence” of the Chimera U.C.S.F software as shown in Supplementary Figure S1A. The quantitative alignment with a graphical color code for root mean squared deviation (RMSD) showed a high similarity index and identity percentage, as corroborated by the similar topology between models (Supplementary Figure S1B).

The Hidden Markov Model software was used to confirm the different transmembrane passes of PS1 based on its primary sequence. Missing fragments are also visible with low probability for a transmembrane pass (Supplementary Figure S2A). The primary sequences for each missing fragment (Met1-Glu72 and Ser290-Glu375) were modeled as a tertiary structure. The model of each fragment was generated using structure predictors Phyre2, I-Tasser, and Quark for folding recognition and structural distribution, using homology comparison algorithms that apply forces derived from primary sequences to the model, and compares them with structures identified experimentally. Posteriorly, models were refined using ModRefiner and Fragment Guided Molecular Dynamics (FG-MD). Resulting structures were aligned with Chimera U.C.S.F, and in order to obtain a structural arrangement from the different models for the two missing fragments, Needleman Wunsch and matrix blosum62 approaches were used. Each of the structure predictors applied a different algorithm of assembly together with homology modeling, protein threading for fold recognition from primary sequences, and assembly without a template, using free modeling *ab initio* taking into account force fields in order to produce the spatial distribution of the models. Subsequently, each model was refined with ModRefiner and FG-MD to improve visualization and analysis of not covalent interactions such as hydrogen bonding, disulfide bridges, hydrophobic, and hydrophilic interactions. The final models obtained with the three different predictive software were validated using QMEAN software for energetic calculations and Procheck software for stereochemical analyses. For Met1-Glu72, the best loop model was obtained with the software Phyre2 (Figure 2B) with an energetic QMEAN6 value of 0,472 in a range between 0–1. The value for Z-score was set similar to the structural size, −1,867. Ramachandran plot of the favorable region was 71.60%, of the allowed region was 20.90%, of the generously allowed region was 4.50%, and of the forbidden region was 3.00%. The best loop model for the fragment Ser290-Glu375 was obtained also with Phyre2, with an energetic QMEAN6 value of 0.455 in a range between 0–1, Z-score with a value of similarity in structural size −2.331. Ramachandran plots of favorable region was 83.3%, of the allowed region was 12.8%, of the generously allowed region was 1.3%, and of the forbidden region was 2.6% (Table 1).

**TABLE 1:** Energetic and stereochemical validation of missing fragments of PS1 protein.

Missing region PS1	Model	Software	QMEAN6	Zscore	Ramachandran plot			
					Favorable region	Allowed region	Generously allowed region	Forbidden region
Met1-Glu72 (M1-E72)	Unrefined	I-TASSER	0.263	−3.379	59.70%	28.40%	4.50%	7.50%
	Refined	FG-MD	0.291	−3.176	62.70%	26.90%	4.50%	6.00%
		ModRefiner	0.235	−3.575	73.10%	22.40%	1.50%	3.00%
Met1-Glu72 (M1-E72)	Unrefined	Phyre2	0.472	−1.867	71.60%	20.90%	4.50%	3.00%
	Refined	FG-MD	0.393	−2.438	58.20%	35.80%	6.00%	0.00%
		ModRefiner	0.232	−3.602	88.10%	7.50%	3.00%	1.50%
Met1-Glu72 (M1-E72)	Unrefined	Quark *ab initio*	0.368	−2.62	84.00%	10.70%	5.30%	0.00%
	Refined	FG-MD	0.286	−3.209	65.70%	20.90%	7.50%	6.00%
		ModRefiner	0.309	−3.041	86.60%	10.40%	1.50%	1.50%
Ser290-Glu375 (S290-E375)	Unrefined	I-TASSER	0.421	−2.606	64.10%	34.60%	1.30%	0.00%
	Refined	FG-MD	0.430	−2.529	65.40%	33.30%	1.30%	0.00%
		ModRefiner	0.445	−2.428	82.30%	17.70%	0.00%	0.00%
Ser290-Glu375 (S290-E375)	Unrefined	Phyre2	0.450	−2.374	60.30%	23.10%	11.50%	5.10%
	Refined	FG-MD	0.449	−2.377	56.40%	32.10%	11.50%	0.00%
		ModRefiner	0.455	−2.374	83.30%	12.80%	1.30%	2.60%
Ser290-Glu375 (S290-E375)	Unrefined	Quark *ab initio*	0.404	−2.74	79.10%	13.90%	7.00%	0.00%
	Refined	FG-MD	0.353	−3.151	53.80%	41.00%	2.60%	2.60%
		ModRefiner	0.376	−2.969	76.90%	17.90%	3.80%	1.30%

According to the results that were calculated from the QMEAN6 energetic parameter in Table 1, Phyre2 was the best structure predicting software for the missing regions (Met1-Glu72 and Ser290-Glu375). This is because it achieved the highest score values in energetic status characterization for the modeled regions compared with the other *ab initio* structure predictors (I-TASSER and QUARK). Lower Z-score value indicated the quality of the models obtained with Phyre2, by assessing the viability of the hypothetical models in relation to structures obtained experimentally that share the same range of values. Finally, the Ramachandran plot showed the distribution of each residue of protein and its dihedral plane with percentages of some residues in the four quadrants of the Cartesian plane i.e., the *x*-axis (Phi) angle and the *y*-axis (Psi) angle. This information was used to validate and assemble the secondary structure of the fragments. We could observe that the Phyre2 models provided high percentage of favorable regions, in this case after refinement. In consequence, the software I-TASSER and Quark *ab initio* generated models (Supplementary Figure S2B) were not selected for further assembly with the rest of the PS1 3D model.

Met1-Glu72 and Ser290-Glu375 fragments generated using Phyre2 were integrated into the PS1 6IYC template as obtained via Cryo-EM. Consequently, a molecular dynamics approach using the VMD/NAMD package was applied to the assembled structure for minimization of all atoms in order to remove any poor initial contacts, to avoid overlapping and to facilitate the fluidity of the model. The resulting full structural model for PS1 in ribbon (Supplementary Figure S3A) and surface density (Supplementary Figure S3B) was then assembled into the γ-secretase model in which the 6IYC template was originally included (Zhou et al., 2019) (Figure 2C). It can be observed that the full hypothetical model for PS1 was not perturbed by the putative N-terminal and loop fragments (Supplementary Figures S3A,B), and that it does not present a structural change in comparison with the original template. In fact, the topology of active sites, Asp257 and Asp385 remain as previously reported (Zhou et al., 2019) (Figure 2C, inset). In consequence, the active pore structure modeled can be further used for the analysis of the structural effects induced by the pathogenic mutations in PS1 that directly modify amino acids in transmembranal domains 6 (TM6) and 7 (TM7). Although a molecular dynamics approach could also be used to evaluate the mobility of the different components of the gamma secretase complex and to assess possible effects of flexible fragments in the active pore, our approach using a hybrid quantum mechanics/molecular mechanics (QM/MM) method allows more sensitivity in the measurement of small topological changes and electronic structural modifications (Van Der Kamp and Mulholland, 2013; Omer et al., 2015; Hofer and de Visser, 2018).

### Functional Analysis of Mutations in the Orthosteric Site TM6

After determining that the structure of the pore is unaffected by PS1 flexible domains, some mutations were selected from the AD mutations database in Alzforum (Alzforum, 2020). Seven different missense mutations located in TM6 close to the active site (Asp257) were selected: Ala246GLu, Leu248Pro, Leu248Arg, Leu250Val, Tyr256Ser, Ala260Val, and Val261Phe. In order to obtain the most sensitive assessment of topological and electronic structure changes generated by these amino acid substitutions, we applied a hybrid QM/MM approach, including the evaluation of electronic potential, ionization potential, and electrostatic surfaces. For the evaluation of polyatomic systems, we chose the force-field Austin Model 1 (AM1), a semi-empirical method for quantum calculations. In this way, we can obtain a description of the modifications in electronic correlations and changes in atomic nuclei topology when comparing wild-type and mutated PS1. Each PS1 mutation induces specific effects in the protein structure. These effects can be on the topology, the electronic surface, or the electrostatic potential. For each mutation, one of these possible changes generates a stronger impact on the structure of the pore, depending on the distance between the amino acid substituted and the active site Asp257.

### Topological Changes Induced by Mutations Ala246Glu, Leu248Pro, and Leu248Arg in PS1

**FIGURE 3:**
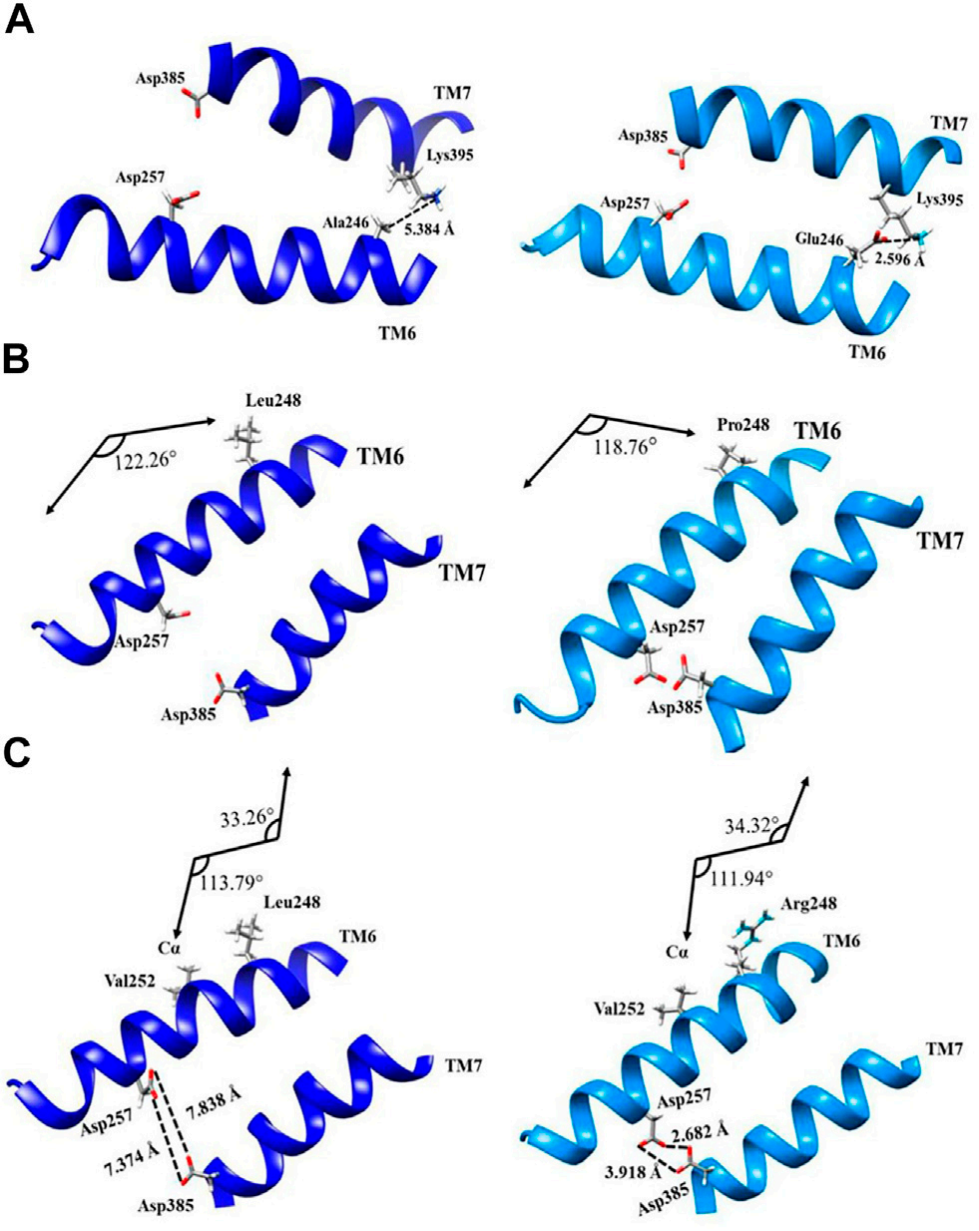
Topological representation of structural changes of PS1 mutations Ala246Glu, Leu248Pro, and Leu248Arg: **(A)** ribbon representation of wild type (left, dark blue) and Ala246Glu mutation (right, light blue) from the interaction with the adjacent α-helix of the transmembrane 7; **(B)** ribbon representation of wild type and Leu248Pro mutation with the kink of α-helix; **(C)** ribbon representation of wild type and Leu248Arg mutation considering the changes in the torsional angles.

Mutations Ala246Glu, Leu248Pro, and Leu248Arg have effects in the chemical properties of the environment of the pore and a direct effect in the secondary structure of the protein in TM6 and TM7. Mutation Ala246Glu presents a chemical change that increases the polarity due to the high electronegativity conferred by adding two oxygen molecules when substituting alanine by glutamic acid. Increased electronegativity induces the formation of transient dipoles, favoring noncovalent interactions, for instance, hydrogen bonding or acid–base reactions with the adequate distances equal or less 2.7 Å (Figure 3A).

In wild-type PS1, position 246 is occupied by alanine, which is not polar and it cannot interact by hydrogen bonding. The lack of polarity in this position brings on London dispersion interactions inside the helix. With the substitution to glutamic acid in this position, the chemical environment changes and TM6 is brought closer to TM7 on this particular location. PS1 amino acid Lys395 presents a basic behavior, and the Ala246Glu mutation facilitates an interaction via hydrogen bonding, facilitated by the decreased distance between TM6 and TM7. In theory, adequate distances to consider for possible adduct formation should be less than 2.7 Å, with the Ala246Glu mutation, the distance between Glu246 and Lys395 is 2.596 Å, while the same distance between wild-type Ala246 and Lys395 is 5.384 Å. The reduced distance between TM6 and TM7 at this point impairs the interaction between the substrate and the orthosteric site. Previous work has shown that drastic changes in polarity for this mutation can favor interactions different than that of the wild type in the diffusion of the carboxy-terminal (CTF99) fragment. This changes the epsilon-cleavage site (ε) of the enzyme and implies a decrease in the total amount of produced peptide. It might also imply an abnormal substrate processing, which follows the cleavages that occur first at Leu-Val (amino acids 49 and 50) or Thr-Leu (amino acids 48 and 49) for the APP substrate (CTF99), thus blocking production up until amino acids 37 or 38 (Funamoto et al., 2019; Funamoto et al., 2004).

PS1 mutation Leu248Pro does not induce major polarity modifications, but it does induce a topological structural change due to the substitution of a leucine to a proline, which contains a ring of five atoms with a nitrogen inside, facilitating a modification in the torsion angle of the helix. The angle between amino acid 248 and the alpha carbon in the side chain of wild-type PS1 is 122,26°, while with mutation Leu248Pro, this angle measures 118,76° (Figure 3B). The effect of these modifications in the torsion of the TM6 helix is similar to the effect observed with mutation Ala246Glu, because it impairs the access of the substrate to the orthosteric site.

PS1 mutation Leu248Arg, on the other hand, modifies polarity in this position. It substitutes leucine, an amino acid with a hydrocarbon side chain, to an arginine, an amino acid with a guanidine group in the extreme of its side chain. The guanidine group contains an electrophilic center, making arginine susceptible to nucleophilic attack by biological systems besides its impact in the amino acid polarity. As with mutations Ala246GLu and Leu248Pro, the distance between TM6 and TM7 decreases. More to the point, the distance between aspartic acids 257 and 385 decreases in the Leu248Arg mutation. The distances of carboxylic groups between aspartic acids 257 and 385 as measured in oxygen atoms sp^2^ and sp^3^ are 7.838 and Å 7.374 Å, respectively. Meanwhile, mutation Leu248Arg decreases these distances to 2.682 Å and 3.918 Å.

As a result of the decreased distance between them, α-helixes of TM6 and TM7 become susceptible to noncovalent interactions, such as hydrogen bonding or electrostatics bonds, making it difficult to access the pore of the substrate. Furthermore, PS1 mutation Leu248Arg also affects the torsion of the TM6 α-helix, producing a kink in the helix. The alteration of the hydrogen bonding pattern modifies the London dispersion interaction between Val252 and the amino acid in position 248 (in the case of this mutation, arginine) turning the helix closer to TM7. In wild-type PS1, with Leu248, the values for these angles are 33.26° and 113.79°, while with the substitution to Arg248 changes them to 34.32° and 111.94°, respectively. The resulting modification of the torsion in TM6 α-helix represents another argument for a plausible blocking of the active site (Figure 3C).

### Electronic Surface Changes Induced by Mutations Tyr256Ser and Ala260Val in PS1

PS1 mutations Tyr256Ser and Ala260Val disturb the topological distribution of electrons in the atoms of affected amino acids. These electronic effects can modify the docking with organic ligands, ions, complex peptides, nucleic acids, dendrimers, and others.

**FIGURE 4:**
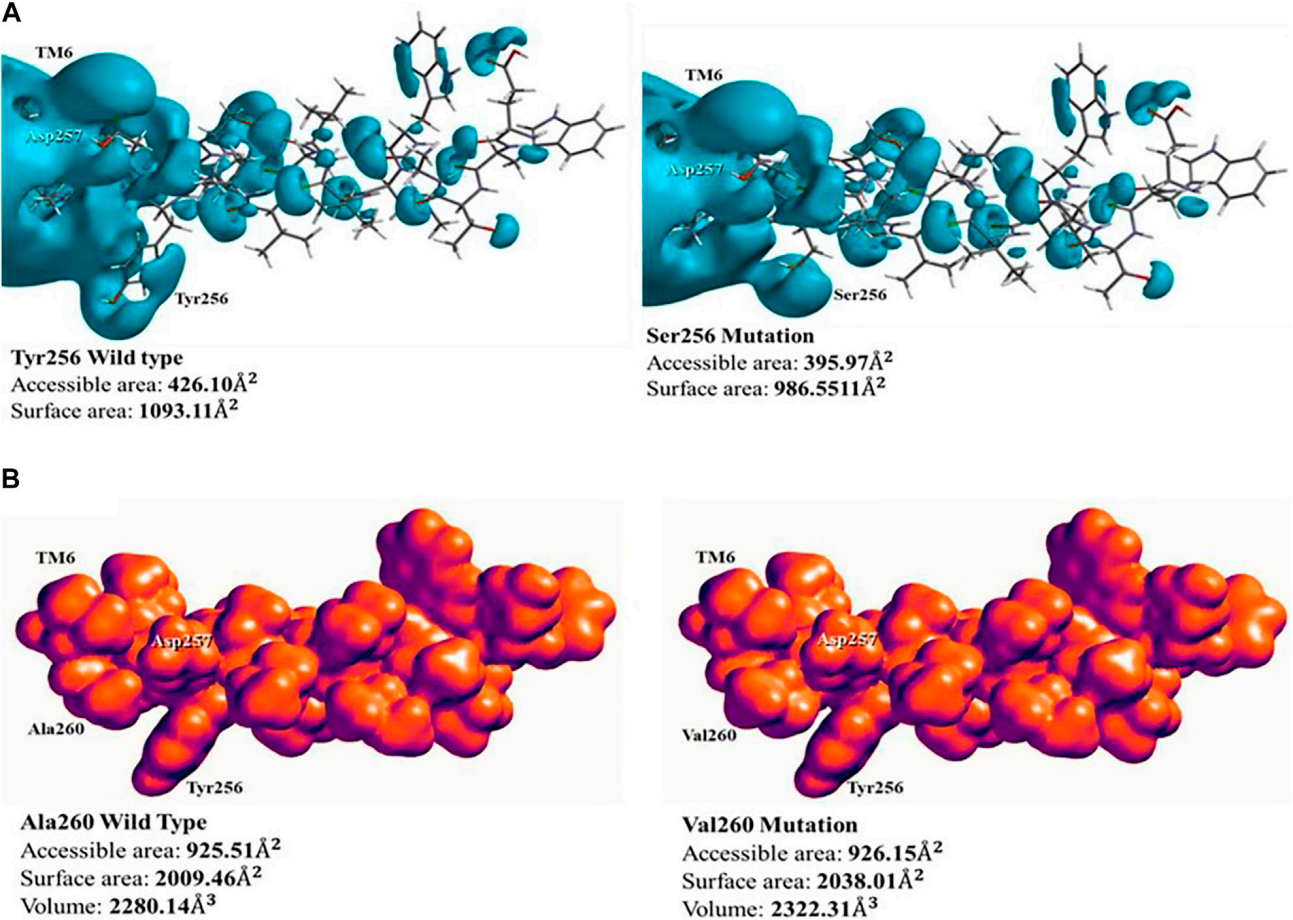
Surface representation of electronic structure of PS1 Tyr256Ser and Ala260Val mutations: **(A)** the potential–potential surface of wild type **(left)** and mutation Tyr256Ser **(right)**. Modification of the surface corresponding to position 256 can be observed; **(B)** density surface of wild type **(left)** and mutation Ala260Val **(right)**, showing the increment in charges volume, blocking potential access to Asp257. The space around Tyr256 is used as a point of reference.

Mutation Tyr256Ser occurs adjacent to Asp257, one half of the active site, indicating that it has a direct effect in the structural conformation and processing of the substrate. In wild-type PS1, the phenol functional group in the side chain of Tyr256 has high acidity, which is consistent with a pKa = 10.06. Besides, amino acid deprotonation is oriented from the phenoxide anion stabilized by resonance. When this amino acid is substituted to Ser256, the side chain of serine contains a hydroxyl functional group, this functional group is less acid than phenol, with pKa = 13.60. Therefore, the hydrogen is not released to the reaction medium. Furthermore, the effect of this substitution in the active site was evaluated on the electronic surface structure with a hybrid QM/MM method. With this approach, a potential–potential surface was created for PS1 TM6 domain. This analysis found that the accessible area of interaction in wild-type PS1, containing tyrosine in position 256, was 426.10 ${\text{Å}}^{2}$ and the total surface area was 1,093.11 ${\text{Å}}^{2}$. With the substitution to serine 256, the accessible area was 395.97 ${\text{Å}}^{2}$ and the total surface area was 986.55 ${\text{Å}}^{2}$ (Figure 4A). Ser256 mutation decreases both areas, and this is an important point to discuss for the possible anchoring of the substrate to the active site. The phenol in the side chain could interact with the substrate by Coulombic interaction with a phenoxide anion or by the effect of the delocalization of electrons in the aromatic ring via stack–stack, stack–cations, or stack–anions interactions. Alternatively, the serine could just interact via hydrogen bonding of the hydroxyl group in the side chain.

PS1 mutation Ala260Val does not present a change of polarity, but it increases the number of carbons in its side chain. The effect of this mutation was measured using a hybrid QM/MM method for the electronic analysis of TM6, by evaluating surface density. In the wild type, with Ala260, the distribution of charges along TM6 has influence on the active site in Asp257 due to its proximity, generating a charges distribution volume of 2,280.14 ${\text{Å}}^{3}$ and a total surface area of 2009.46 ${\text{Å}}^{2}$. With the substitution to Val260, there is a modification on the distribution of charges with a distribution volume of 2,322.31${\text{Å}}^{3}$ and a total surface area of 2038.01 ${\text{Å}}^{2}$ (Figure 4B). Taking into account these values, the increase in distribution volume and surface area could be a result of the increased number of carbons, besides the inclusion of a methyl group due to the substitution to valine. Therefore, there is a change in the intrinsic distribution of electronic density in front of the active site Asp257, blocking the access and possible interaction with the substrate in the structural model.

### Modifications in the Electrostatic Potential Map in PS1 Mutations Leu250Val and Val261Phe

**FIGURE 5:**
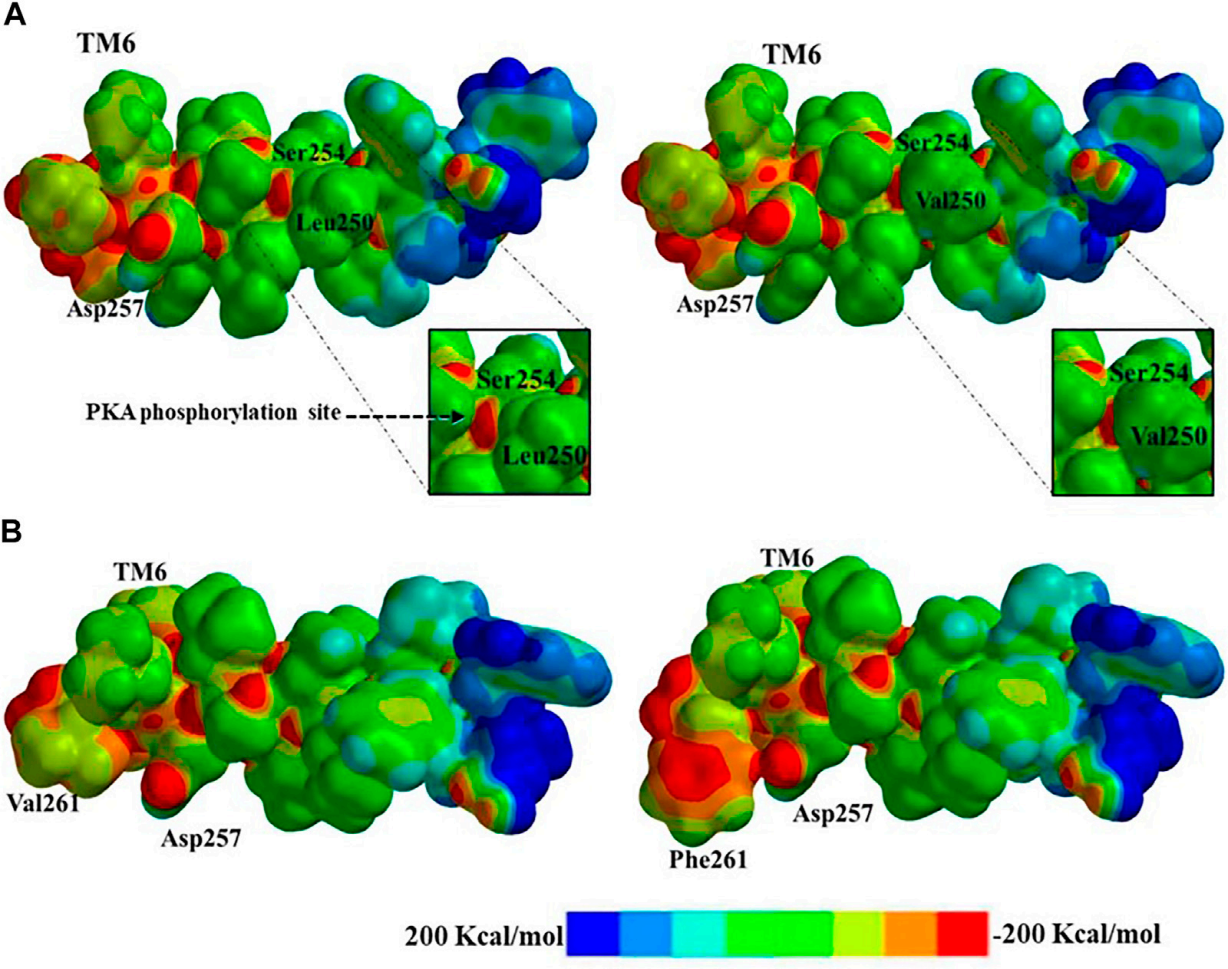
Electrostatic potential map representation: **(A)** electronic distribution and phosphorylation blockage of wild type **(left)** and PS1 mutation Leu250Val **(right)** to putative protein kinase A (PKA) phosphorylation; **(B)** electronic distribution and resulting steric clash of the substitution from wild type **(left)** to PS1 mutation Val261Phe **(right)** due to the aromatic electronic effect in the possible interactions with the substrate. Electronic distribution within the range of −200 Kcal/mol to 200 Kcal/mol is represented by an eight-color scale.

Aside of topological effects or changes in surface area or density, other possible effects of PS1 mutations could be in the electronic distribution within the electrostatic potential. The PS1 Leu250Val mutation does not present polarity changes given that both amino acids (Leu and Val) do not present any polar feature (London dispersion), the main difference between amino acids is one extra carbon in the structure of valine, and this implies a possible spatial effect, given that the hydropathicity is the same. Using the ProtScale software, we quantified the hydrophobic and hydrophilic forces with the Kyte and Doolittle approach. In this position in PS1, the wild-type Leu250 has a hydropathic index of 2,522, while the substituted Val250 presents a hydropathic index of 2,567, with similar polarity behavior (Supplementary Figure S4). Therefore, there are no topological modifications in the α-helix, given that the similarities in hydrophobicity do not affect the London dispersion forces. Likewise, surface electronic density analysis did not detect changes between the wild type and the mutation. However, there was the option to evaluate the electrostatic potential map using Spartan 18.0 software. In effect, there is a modification in the electronic structure in the vicinity of the mutation site, reducing the access to Ser254 in the Val250-mutated PS1 (Figure 5A). This modification can have a direct impact in the functionality of the protein. We searched for possible affected interactors using the software XPASY and the tool NetPhosK 2.0. We found that Ser254 is a phosphorylation site for kinase PKA in the wild type situation, with a score 0.50 (Figure 4A, insets). In conclusion, when the Val250 substitution takes place, position Ser254 is blocked with the hydrocarbon side chain and its high electronic density site cannot be docked by PKA, resulting in loss of the phosphorylation site.

We did not find experimental reports in the literature that confirm phosphorylation for this position in PS1. However, this protein is highly phosphorylatable, and it has associated functions such as cell signaling, Ser346 being a recognition motif for caspase in apoptosis regulation (Fluhrer et al., 2004). We have also assessed the differential effect of phosphorylation of the A246E mutation in the PS1 transmembrane domain and the N141I mutation in PS2. This has been done considering that these mutations could impact phosphorylation due to its structural localization. However, no differences have been found in the effect of PS1 and PS2 phosphorylation. Likewise, many remaining available phosphorylation sites have been proposed. These remain after the γ-secretase enzyme carries out substrate proteolytic processing. This leads to a structural change in the enzyme that renders amino acids accessible in cases where they were initially inaccessible upon phosphorylation of casein kinase 1 and 2, or of PKA and PKC (Walter and Haass, 2010; Walter et al., 1997). The position for the phosphorylation site is currently proposed to be serine 367, as it has been found to be closely related to the dynamics of microglia development and has also been found to have a protective function. This encourages autophagosome–lysosome assembly, which increases the degradation of β-CTF99 carboxi-terminal, thereby decreasing amyloid peptide synthesis (Ledo et al., 2020; Bustos et al., 2017).

The last PS1 mutation evaluated is Val261Phe, and here we can observe the change in the aliphatic side chain to an aromatic group with a direct effect on the structure of TM6. Phenylalanine has a high electronic density due to the aromatic ring, and the resulting increased electron density blocks the active site Asp257. The electrostatic map represents the potential and electronic distribution in the range of -200 Kcal/mol to 200 Kcal/mol. In the wild-type PS1, Val261 shows relatively low electronic density, while the Phe261 mutant presents a wider area with higher electronic density with a score of −100 to −150 Kcal/mol (Figure 5B). Furthermore, this substitution has an effect in the topology. The aromatic group produces a change in the dihedral angle due to the hybridization of the aliphatic and aromatic carbons in the structure, reducing distances for bonding via steric clash. The angles between the α carbon and the lateral side chain are modified, affecting the structure and the dihedral angle manifest differences in the topological representation with angles of −60.47° for wild type and −132.04° for mutated PS1 Val261Phe. The dihedral changes induce a kink in the α-helix of TM6 and the substrate can be hindered when entering the pore (Supplementary Figure S5).

**FIGURE 6:**
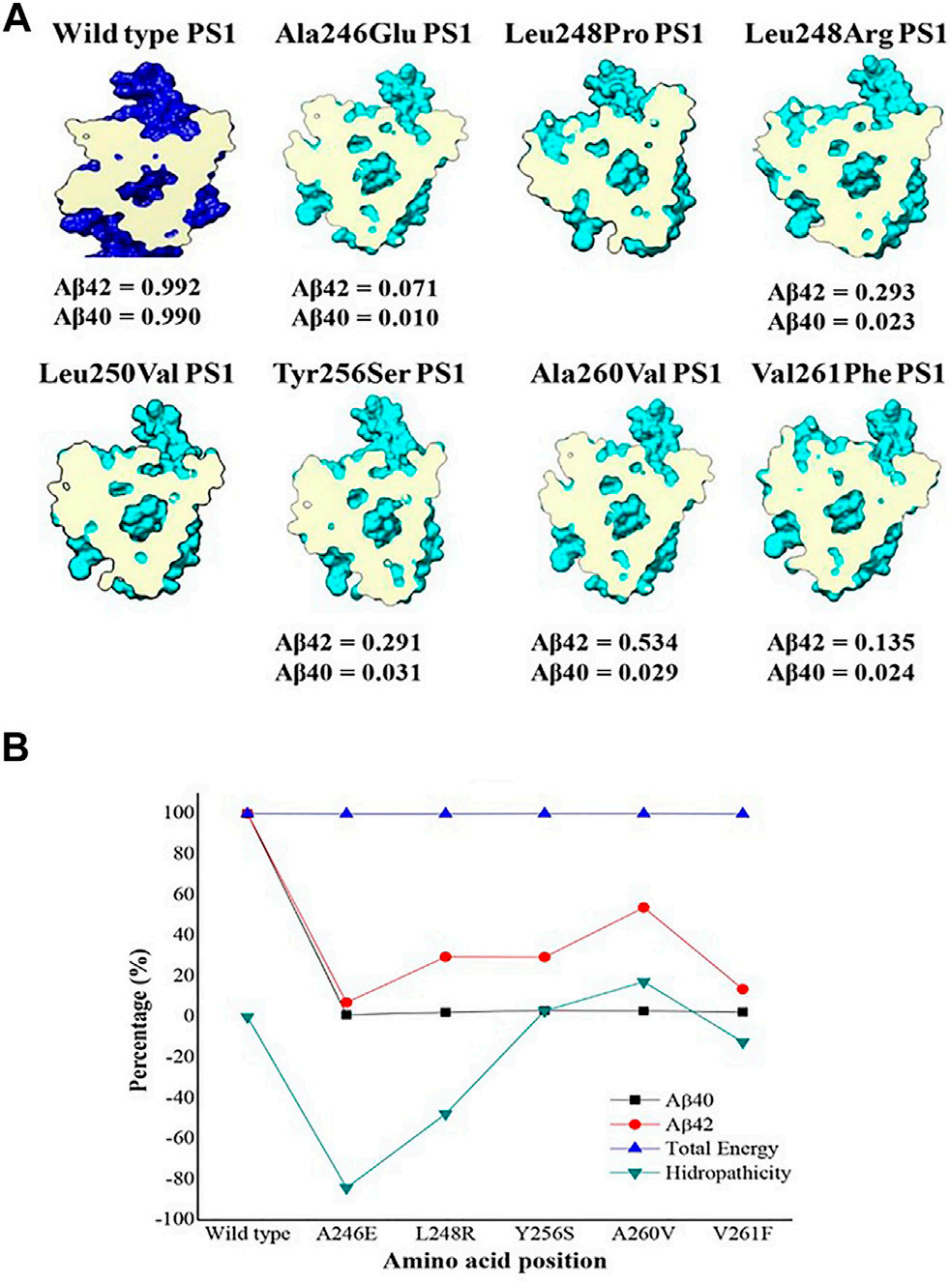
Top view section of wild-type PS1(dark blue) and the seven mutations analyzed in TM6 (pale blue) with a cross section at the same level of the catalytic pocket (magenta). **(A)** β ratio values were obtained from reference (Sun et al., 2016); **(B)** Energy comparison plot, hydrophobicity and total amount of amyloid peptide.

In summary, the consequences of a variety of structural and electronic modifications in the active domains of PS1 as a result of point mutations suggest a possible functional effect in the catalytic activity for the processing of APP as a substrate. This effect could be considered as loss of function given that experimental data from the studied mutations show a decreased production of both Aβ 1–40 and Aβ 1–42 together with increased Aβ 1–42/1–40 ratio (Sun et al., 2016) (Figure 6A and Supplementary Table S1). Besides, the evaluation of topology, surface area, volume, and electrostatic potential is necessary to understand the structural behavior of PS1. These modifications can be summarized by a top view section in the upper plane of the protein. Due to the combination of the structural effects, the shape of the pore defined by the space and distance between TM6 and TM7 is noticeably modified in PS1 mutants. In some cases, the apparent volume and shape of the pore are radically different, hinting to a possible effect in the accessibility of the pore by the substrate. For instance, in mutation Ala260Val, the structure of the pore is severely modified, and the production of Aβ 1–40 is depleted, while the production of Aβ 1–42 is half of that on the wild type (Sun et al., 2016), hinting to the accessibility effect mentioned above (Figure 6A). The energetic calculation is obtained for the entire system with the subtraction formula in quantitative terms and considering the catalytic pocket in the multiscale model, thus obtaining the results reported in Table 2.

**TABLE 2:** Energetic values calculated in the system with PS1 in the γ-secretase enzyme.

Protein PS1	Energy MM (Full length)	Average MM (Full length)	Energy MM (QM)	Average MM (QM)	Energy QM	Average QM	Total Energy: EQM + EMM-EMM(QM)
Wild type	2962167.1071	2962167,108 (+/−) 0,017	−40.6771	−40,67710 (+/−) 0,00040	−5104.5531	−5104,5604 (+/−) 0,0067	2957103,225(+/−) 0,023
	2962167.0920		−40.6775		−5104.5661		
	2962167.1261		−40.6768		−5104.5621		
Ala246Glu	2961014.1399	2961014,14 (+/−) 0,89	−161.5506	−161,5503 (+/−) 0,0021	−5534.4918	−5540,2 (+/−) 4,9	2955635,5 (+/−) 5,9
	2961015.0300		−161.5481		−5543.0927		
	2961013.2500		−161.5523		−5543.0906		
Leu248Pro	2964064.3892	2964064,39 (+/−) 0,16	772.9771	772,97740 (+/−) 0,00090	−4998.3374	−4998,337 (+/−) 0,012	2958293,07 (+/−) 0,17
	2964064.2220		772.9784		−4998.3491		
	2964064.5480		772.9768		−4998.3252		
Leu248Arg	2959517.4753	2959517,48 (+/−) 0,05	−13.3939	−13,3937 (+/−) 0,0016	−4677.5868	−4672,0 (+/−) 9−6	2954858,8(+/−) 9,7
	2959517.4220		−13.3952		−4677.5844		
	2959517.5312		−13.3921		−4660.9302		
Leu250Val	2962020.7393	2962020,7398 (+/-) 0,0090	108.0198	108,01970 (+/−) 0,00050	−4676.2334	−4676,2318 (+/−) 0,0025	2957236,488 (+/−) 0,011
	2962020.7310		108.0192		−4676.229		
	2962020.7492		108.0202		−4676.2331		
Tyr256Ser	2961819.2446	2961819,24 (+/−) 0,19	30.3606	30,36050 (+/−) 0,00040	−5235.8072	−5235,47 (+/−) 0,58	2956553,41 (+/−) 0,77
	2961819.0530		30.3601		−5234.7973		
	2961819.4348		30.3609		−5235.809		
Ala260Val	2964306.3948	2964306,40 (+/−) 0,32	100.4126	100,41260 (+/−) 0,00050	−5168.3129	−5164,3 (+/−) 11,7	2959041,7 (+/−) 12,0
	2964306.7205		100.4121		−5173.4372		
	2964306.0722		100.413		−5151.0903		
Val261Phe	2960691.6238	2960691,624 (+/−) 0,090	160.2564	160,25730 (+/−) 0,0010	−4955.9667	−4956,28 (+/−) 0,53	2955575,09 (+/−) 0,62
	2960691.5340		160.2571		−4955.9701		
	2960691.7131		160.2583		−4956.8911		

By analyzing the results in (Figure 6B), it is determined from an energetic standpoint that due to several mutations, there is not a significant change at an energetic level. However, there is a decreased size of the catalytic pocket, which leads to the enzymatic function being affected. This explains the decrease in the total amount of peptide for Aβ 1–40 and Aβ 1–42. However, the change of amino acid for mutations is not synonymous if they show an effect on hydrophobicity. Therefore, certain cuts of amyloid-β are favored, which is reflected in the cut ratio at the experimental level with the Aβ42/Aβ40 peptide proportion. As a result, an analysis focused on the lateral chain is validated with changes in topology and electronic structure, as was previously shown. The graph reveals that the increase in hydrophilicity results in the cleavage route that leads to producing Aβ42 peptide instead of Aβ40. This is because it is the most commonly found peptide in the amyloid plaques and the graphical trend of its hydrophobicity is very similar to the data that were experimentally reported on the peptide cuts of 42 amino acids. Likewise, the γ-secretase enzyme with mutations and with these changes in polarity profiles favors certain processing routes that can produce the most frequent amyloid peptide fragments. In addition, due to the mutations, enzyme activity could be modified and could encourage cleaving up to the Aβ38 and Aβ37 amyloid peptide fragments. These fragments are frequently found in senile plaques but are not as pathogenic as Aβ 40 or Aβ 42, which tend to undergo oligomerization more readily (Murakami et al., 2003; Chen et al., 2017; Morel et al., 2018; Song et al., 2018).

Previously, a molecular dynamics approach by Chávez-García et al. was used to analyze the effects of PS1 mutations in the catalytic domain of the protein (Chávez-García et al., 2019), (Aguayo-Ortiz et al., 2017). Briefly, their approach involved amino acid network and protonation analysis for thirteen PS1 mutations via all-atom molecular dynamics simulation. Among their findings, an increased number of correlations for different mutations were identified. Interestingly, two of the mutations analyzed by this approach are localized in TM6 and were also evaluated in the present work (Ala260Val and Val261Phe). They found that these two mutations presented increased number of correlations and they also suggest that the amino acid substitution might affect the entry gate (Chávez-García et al., 2019). We consider that our approach brings a different view of the problem, and that both approaches (molecular dynamics and hybrid QM/MM) are valid and complementary when analyzing the effect of mutations in structural protein chemistry.

## Conclusion

Protein functional studies through structural modeling in neurodegenerative diseases is a useful approach for understanding the effect of some genetic variants that translate in specific protein modifications. In the case of PS1, it opens a window to understand how its structure affects its function and those of the γ-secretase complex and its four subunits. Given that PS1 has two flexible domains that have not been resolved satisfactorily via experimental approaches, we have developed a model using structure prediction software. Flexible regions present experimental challenges for protein structure studies, such as their low electron-dense zone with low signal emission that results in low structural resolution for Cryo-EM studies or affecting crystallization for X-ray analysis (Bai et al., 2015; Rossi et al., 2012; Heo et al., 2017). Therefore, an *in silico* approach seems to be the best alternative for resolving the full structure of PS1, until further experimental models are obtained. Homological modeling, using assembly by threading and reconstruction *ab initio*, was used to create a hypothetical construct for the missing fragments with their respective energetic and stereochemical characterization. Our approach included algorithms of molecular dynamics methods that consider force fields and the primary sequences of amino acids in the construction of proteins. The completed model for PS1 was then useful to assess possible effects of the flexible domains in the pore. In our reconstructed model, we observed that the predicted assembly for both flexible fragments did not affect the topology and the connectivity matrix of the most current template for PS1 (Zhou et al., 2019) and did not affect the structure of the pore constituted by TM6 and TM7 (Supplementary Figure S3). It is possible that PS1 pathogenic mutations localized in the flexible pores affect pore accessibility by other means different from direct modifications on the active site.

Additionally, we analyzed seven PS1 mutations localized in TM6 and in the proximity of Asp257, in order to assess the direct effect of these mutations in the active site. The structural changes were assessed using a topological approach for distance variations, torsion angles, and dihedral angles and electronic changes with the distribution of charges and surfaces in the system. Macromolecular systems present a problem for structural biology, such as their polyatomic constitution and the high number of possible multiple interactions. In these cases, hybrid QM/MM methods are useful for the study of polyatomic systems given that they assess the interaction of the electronic structure with a stochastic measure of energy and optimization of structural conformers (Murphy et al., 2000; Silva et al., 2015; Zou et al., 2017).

For PS1 mutations Ala246Glu, Leu248Pro, and Leu248Arg, topological changes, such as the modification of distances between TM6 and TM7 as a result of changes in the kink of the TM6 helix, seemed to be the most relevant for their possible effect in the active site. On the other hand, PS1 mutations Leu250Val, Tyr256Ser, Ala260Val, and Val261Phe produce more noticeable modifications in the electronic structure of TM6, affecting the electronic surface, charge distribution volume, and electrostatic potential, finally blocking the access of the substrate. Interestingly, the modification of the electronic distribution for Ser254 elicited by PS1 mutation Leu250Val has a direct effect in the corresponding phosphorylation site with possible functional repercussions. Independently of the specific change, all the studied mutations affected the shape of the pore, possibly affecting the accessibility of the substrates to the active site or affecting the kinetics of its processing. There are experimental data for Aβ processing of five out of the seven PS1 mutations we analyzed. All of them show decreased production of Aβ in comparison with the wild-type enzyme, with some of them increasing the relative production of Aβ 1–42 (Sun et al., 2016), perhaps as an effect of major changes in the pore (Figure 6A).

We consider that the use of QM/MM hybrid methods might be an ideal approach for the study of single-point mutation effects in macromolecular systems as complex as that of γ-secretase and PS1. With the development and access to more powerful computational systems, this kind of studies will provide a wide array of possibilities for functional analysis and the development of better targeted drug design.

## Data Availability

The datasets presented in this study can be found in online repositories. The names of the repository/repositories and accession number(s) can be found in the article/Supplementary Material.
