# Supplementary material for: Protein Predictive Modeling and Simulation of Mutations of Presenilin-1 Familial Alzheimer’s Disease on the Orthosteric Site
Source: Front Mol Biosci. 2021 Jun 2;8:649990. doi: 10.3389/fmolb.2021.649990 (PMC8206637; doi:10.3389/fmolb.2021.649990)
Supplement: Supplementary file 1 [file DataSheet1.PDF]

Supplementary Material

Protein predictive modeling and mutations simulation of presenilin-1 familial Alzheimer's disease on the orthosteric site.

Alejandro Soto-Ospina[1,3], Pedronel Araque Marín[2*], Gabriel Bedoya[1], Diego Sepulveda-Falla[3,4] and Andrés Villegas Lanau[1,3*]

[1]University of Antioquia, Faculty of Medicine, Group Molecular Genetics, Medellín, Colombia.

[2]EIA University, School of Life Sciences, Research and Innovation in Chemistry Formulations Group, Envigado, Colombia

[3]University of Antioquia, Faculty of Medicine, Group Neuroscience of Antioquia, Medellín, Colombia.

[4]Molecular Neuropathology of Alzheimer's Disease, Institute of Neuropathology, University Medical Center Hamburg-Eppendorf, Hamburg, Germany.

***To whom correspondence should be addressed:**

Pedronel Araque Marín, e-mail: pedronel.araque@eia.edu.co

Research and Innovation in Chemistry Formulations Group, School of Life Sciences, University EIA, Via José María Córdova airport, Postal Code 055428, Envigado, Colombia

Andrés Villegas Lanau, e-mail: andres.villegas@udea.edu.co

University of Antioquia, Faculty of Medicine, Group Molecular Genetic (GenMol), Calle 62 #52-59 Laboratory 411-412, Medellín - Antioquia, Colombia.

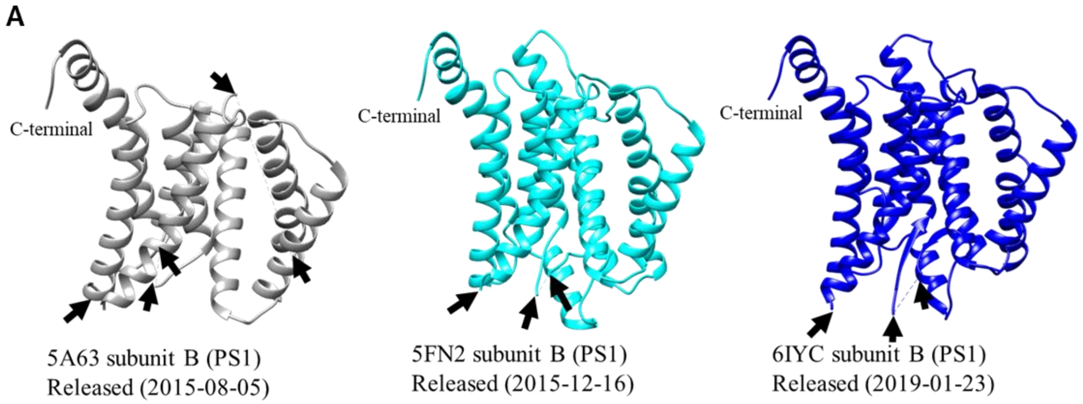

Template ID (PDB)	Missing areas (Amino acids)
5A63 Subunit B	1-76; 108-165; 261-377.
5FN2 Subunit B	1-77; 289-377.
6IYC Subunit B	1-72; 290-375

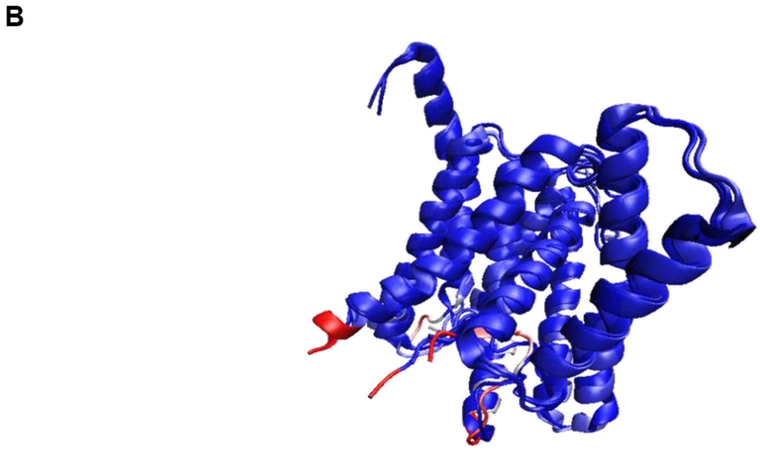

Alignments	QH	RMSD	Identity percentage
Template 6IYC/5FN2	0.7287	1.3928	82.54
Template 5FN2/5A63	0.5818	1.1607	67.97
Template 5A63/6IYC	0.5373	1.1644	66.14

Supporting Figure 1. *Models and characterization of the catalytic subunit of PS1 with C-Terminal and missing N-Terminal fragment: A) Proposed experimental electronic microscopy structure of protein PS1 with missing fragments in black arrows; B) Quantitative alignment of protein PS1 according to the values of high standard deviation (RMSD) in the alignment are represented in (Red), standard deviation in a range between [2.0-2.5] Å of standard deviation in (White), highly conserved values and low standard deviation (Blue).*

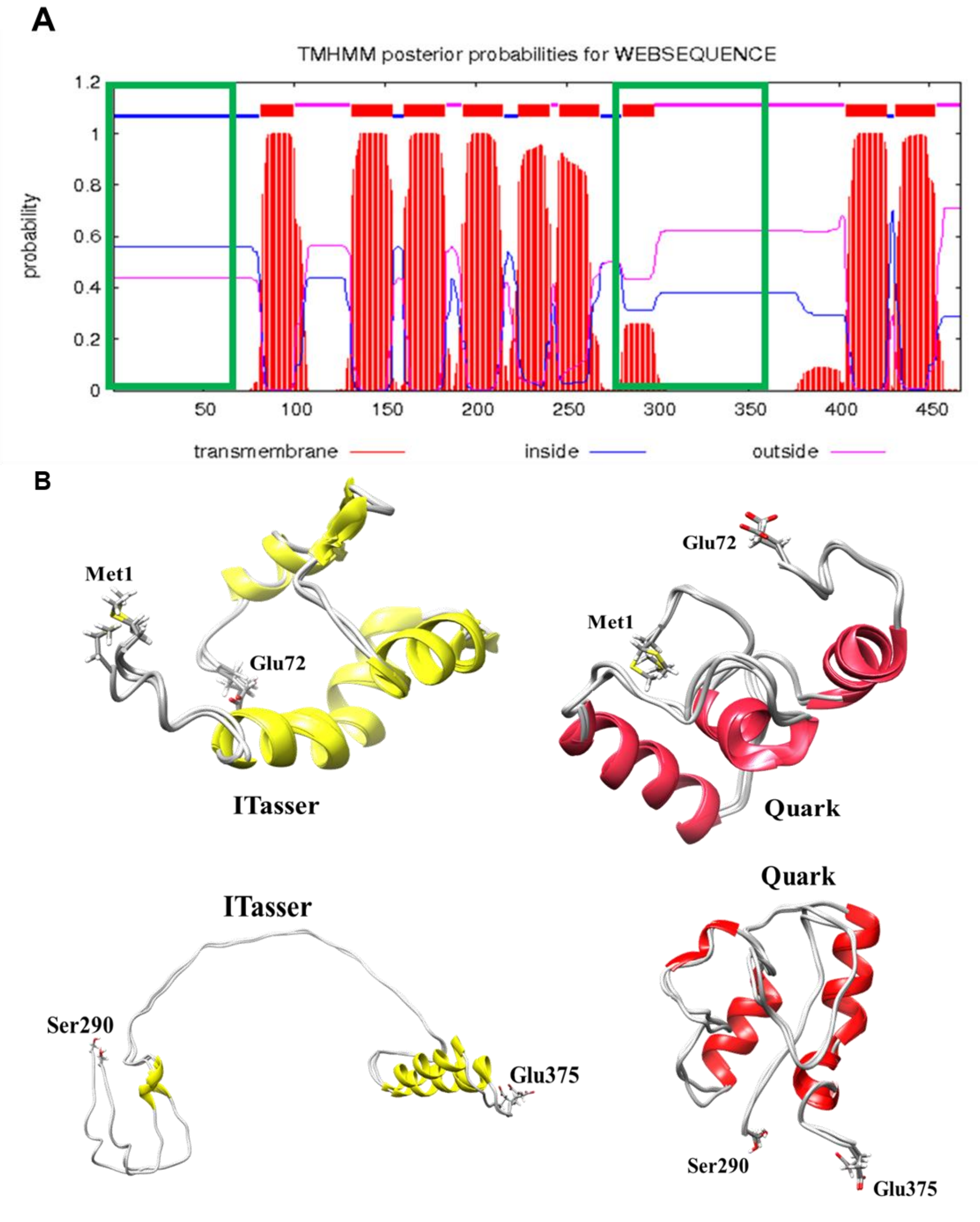

Supporting Figure 2. *Computational representation and hypothetical construction of PS1 models: A) Probability graph of protein PS1 for the transmembrane location with Hidden Markov Model Transmembrane (Green inset represent the zone with missing fragments of all model); B) Fragments in the prediction of Met1-Glu72 and Ser290-Glu375 amino acids by predictors of structure I-Tasser and Quark.*

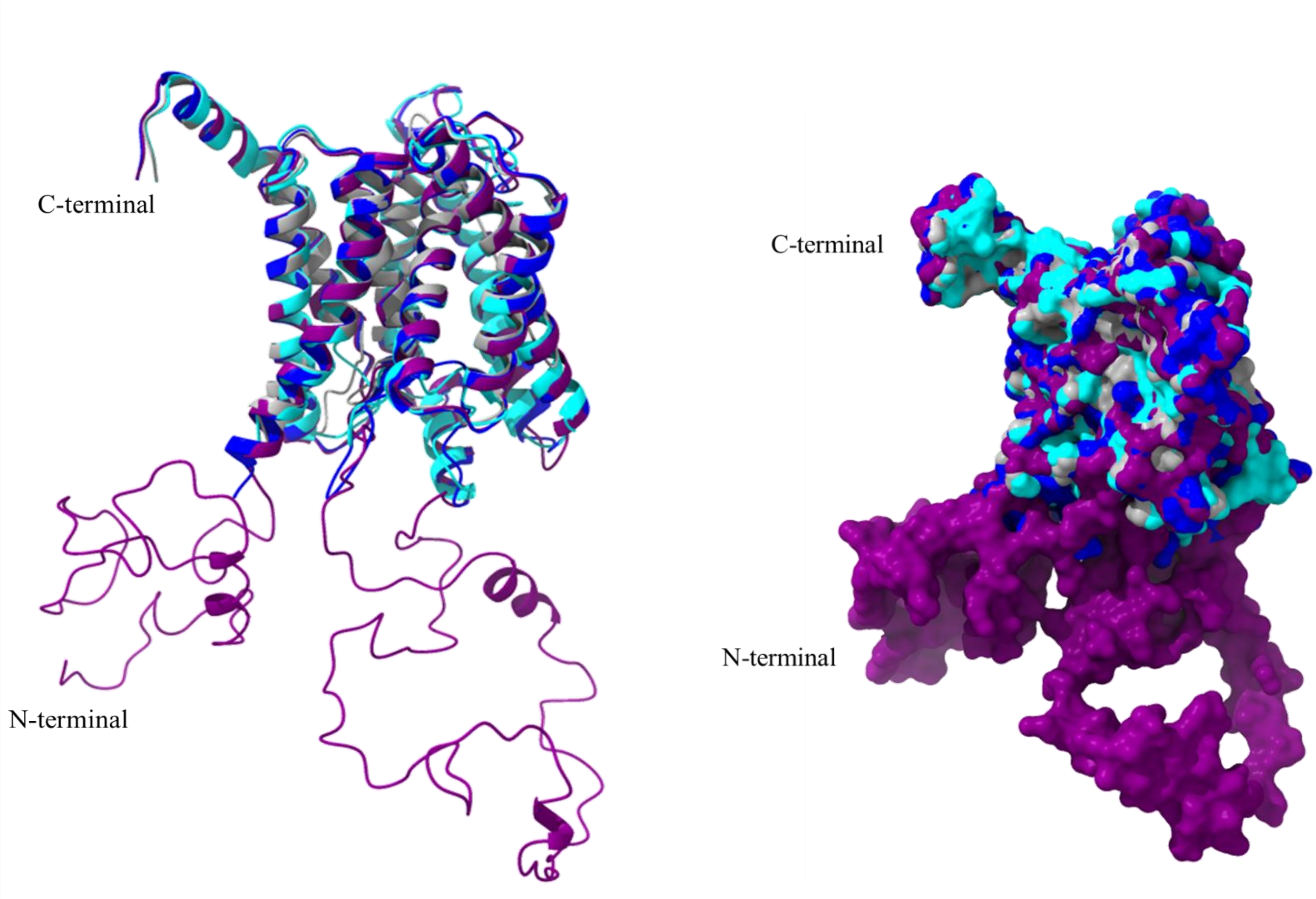

A **B**

Supporting Figure 3. *Structural models of PS1 protein and tridimensional alignment Needleman-Wunsch:A) Ribbon representation of hypothetical model of PS1 (purple) and reported templates of PS1 with ID: 5A63 (Dark-grey), 5FN2 (Cyano) and 6IYC (Dark blue);B) Density surface of the alignment with the same colors.*

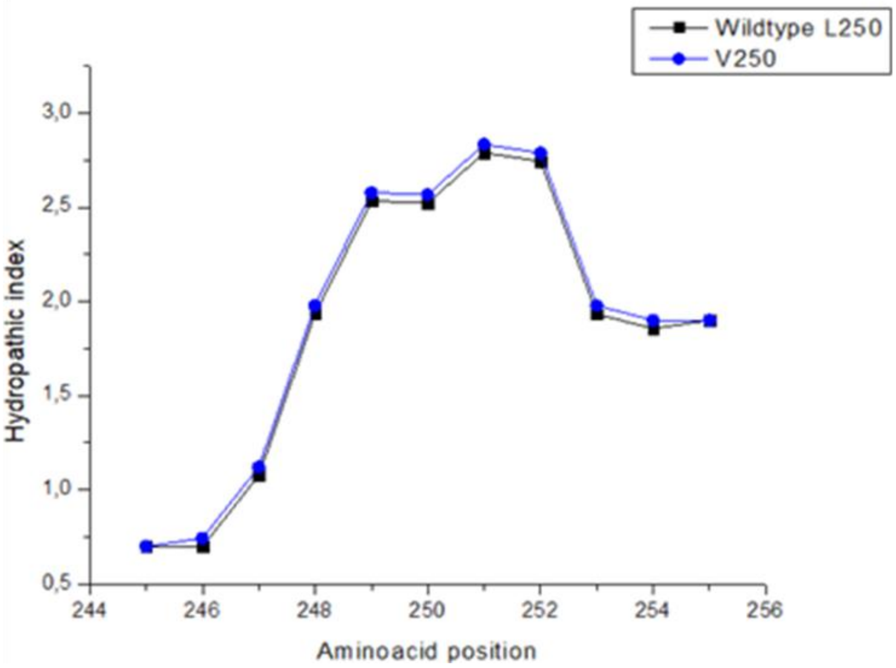

Supporting Figure 4. *Hydropathic index curves for wildtype PS1 and mutation Leu250Val. Note the similarity between wildtype and mutant proteins.*

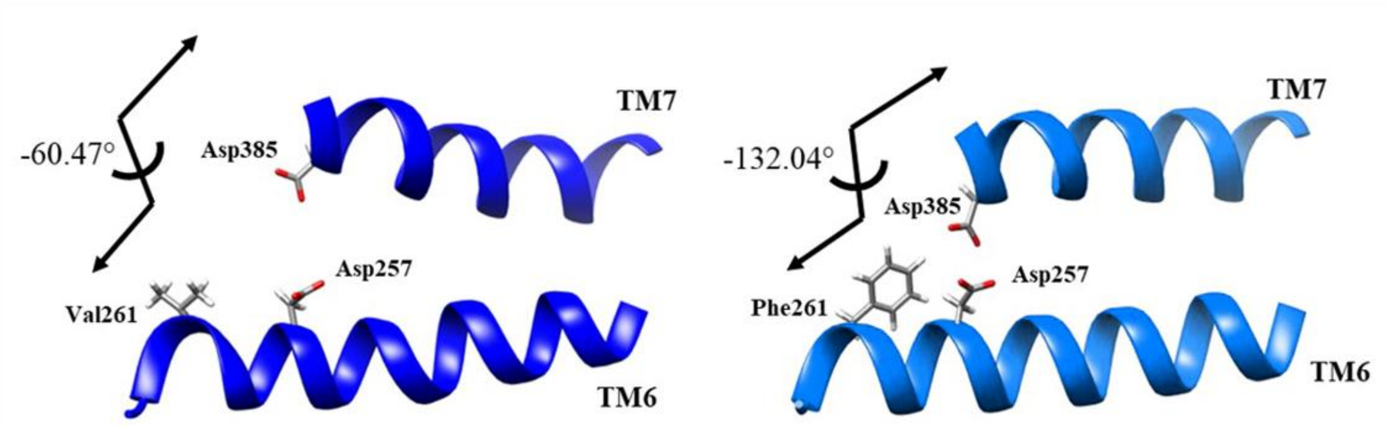

Supporting Figure 5. *Topological representation of structural changes of PS1 mutation Val261Phe. Dark blue ribbons represent PS1 wild type (left) and light blue ribbons represent Val261Phe mutation (right), from the interaction with the adjacent α-helix of the transmembrane 7 also considering the changes in the torsional angles.*
